# Heat-related illness symptoms and mental health in Florida farmworkers

**DOI:** 10.3389/fpubh.2026.1797619

**Published:** 2026-05-18

**Authors:** Andrea Daniela Castellano, Rodrigo Puentes, Ernesto Ruiz, Iliana Yamileth Rodriguez, Vicki Stover Hertzberg, Linda A. McCauley, Roxana C. Chicas

**Affiliations:** 1 University of Pennsylvania School of Nursing, Philadelphia, PA, United States; 2 Gangarosa Department of Environmental Health, Rollins School of Public Health, Emory University, Atlanta, GA, United States; 3 Farmworker Association of Florida, Apopka, FL, United States; 4 Emory University Emory College of Arts and Sciences, Atlanta, GA, United States; 5 Emory University Nell Hodgson Woodruff School of Nursing, Atlanta, GA, United States

**Keywords:** anxiety, depression, farmworkers, heat-related illness (HRI), mental health, occupational health

## Abstract

**Background:**

Farmworkers face disproportionate occupational heat exposure and high rates of heat-related illness (HRI), and emerging evidence suggests associations between heat exposure and adverse mental health outcomes.

**Objective:**

This exploratory pilot study aimed to (1) characterize the burden of depression, anxiety, and climate change psychological distress among Florida farmworkers relative to national benchmarks, (2) explore cross-sectional associations between HRI symptoms and mental health outcomes, and (3) examine cross-sectional associations between perceived impacts of Florida Senate Bill 1718 (S.B. 1718) and mental health outcomes.

**Methods:**

In July 2024, 53 adult farmworkers (58% female, 42% male) were recruited through the Farmworker Association of Florida (FWAF) in Apopka and Pierson, Florida. Community health workers verbally administered the PHQ-9, GAD-7, CCPD scale, an eight-symptom HRI checklist, and two investigator-developed items on S.B. 1718. Workday heat index was derived from the Florida Automated Weather Network. Analyses included descriptive statistics, Fisher’s exact tests, and linear and logistic regression adjusted for age, sex, and agricultural tenure.

**Results:**

Moderate-to-severe anxiety, depression, and climate change psychological distress were observed in 21, 25, and 34% of participants, respectively, exceeding U.S. adult and Hispanic-adult benchmarks. Cross-sectional associations were observed between the number of same-day HRI symptoms and each mental health outcome (all *p* < 0.05 unadjusted). Participants reporting feeling discouraged from seeking hospital care due to S.B. 1718 had higher mean PHQ-9 scores than those who did not (6.59 vs. 3.00; *p* = 0.008). Confidence intervals around regression estimates were wide, reflecting sparse-data instability rather than precise effect sizes.

**Significance:**

This study addresses an empirical gap at the intersection of occupational heat exposure, immigration policy, and mental health among farmworkers in the southeastern United States. The findings identify mental health as an under recognized dimension of farmworker occupational health and point to the need for integrated approaches that link heat-safety protections, routine mental health screening in community-based settings, and attention to the policy environments that shape healthcare access. Larger, longitudinal studies are needed to confirm these associations and to guide the scale and design of such integrated responses.

## Introduction

Agriculture is a climate-sensitive industry in which farmworkers experience disproportionate physiological burdens, including heat-related illness (HRI) and elevated core body temperatures, and face the highest rates of heat-related mortality among occupational groups (1–3). HRI refers to a spectrum of conditions caused by environmental heat stress that overwhelms the body’s thermoregulatory capacity, ranging from early manifestations (e.g., cramps, heat syncope, dizziness and nausea) through heat exhaustion to heat stroke with multiorgan failure (4). Within the United States (U.S.), there are an estimated 2.9 million farmworkers, 78% of whom identify as Hispanic (5). Women comprise roughly 25% of the agricultural workforce (6). Agriculture is a major industry in Florida, estimated to have 150,000 farmworkers, predominantly immigrant workers who work in high humidity and hot temperatures (7). Studies of Florida farmworkers have reported samples in which approximately half or more of participants are female (2, 8). The majority of hired farmworkers in the U.S. are foreign-born, predominantly from Mexico and Central America, and work in labor-intensive agricultural jobs characterized by long hours and low wages (6). Approximately half of farmworkers in the U.S. lack health insurance, most have limited English proficiency, and the average educational attainment is ninth grade (6). Furthermore, farmworkers often live in rural areas with shortages of health and mental health care providers (9).

While several studies have documented health disparities among farmworkers, less is known about the mental health status of farmworkers (10). Additionally, most mental health studies in the agricultural industry have focused on farmers (owners of farms and ranches), with fewer studies evaluating the mental health status of farmworkers (laborers) (11, 12). This gap is concerning given that farmworkers, a workforce primarily composed of Hispanic immigrants, may be at higher risk for mental health conditions, particularly depression and anxiety, and even lower utilization of mental health services (10, 13). Among Hispanic communities in the United States (U.S.), mental illness is on the rise (14), with depression being the most prevalent mental health condition, followed by anxiety. Several factors have been linked with an increased risk of depression and anxiety among Hispanic communities, such as acculturation, precarious immigration status within families, and psychosocial stress due to food and housing insecurity (15–17). Low utilization of mental health services among Hispanics has been attributed to language barriers and a shortage of culturally competent mental health providers (18–20) as well as cost and lack of health insurance (18, 21). The National Center for Health Statistics reports that 16% of Hispanic adults experience any symptoms of anxiety and 19% experience any symptoms of depression (22).

Despite the well-documented physical health burden of agricultural work, the relationship between chronic occupational heat exposure and mental health among farmworkers remains poorly understood. The 2016 U.S. Global Change Research Program Exposure Pathway framework identifies mental health outcomes, alongside HRI, as plausible health consequences of climate-driven occupational heat exposure (23). However, research on mental health impacts of climate change has focused primarily on natural disasters, such as hurricanes, earthquakes, and wildfires, which may cause trauma and post-traumatic stress disorder through displacement, loss of income, and loss of family members (24). More recently, terms such as climate anxiety, eco-anxiety, and solastalgia have emerged to describe the psychological responses resulting from gradual, long-term environmental changes due to climate change (25–27). These constructs describe psychological responses to awareness of climate change as an abstract environmental concern and are conceptually distinct from the direct physiological stress of chronic bodily exposure to occupational heat. Separately, ecological studies have reported that thermal discomfort from heat exposure worsens emotional states (25, 28). There has been increased awareness of climate change’s role in occupational safety and growing calls to better understand climate-related mental health impacts among workers (29).

In our prior studies with farmworkers in Florida, workers have reported experiencing HRI symptoms during their work shifts, such as headaches, dizziness, nausea, and muscle cramps (3, 8, 30). We have also documented elevated core body temperatures and acute kidney injury associated with heat index (2, 31). However, we have not assessed the relationship between heat exposure and working conditions to their mental health. The present pilot extends this program of research to mental health outcomes not previously measured in our studies. The mental health instruments, HRI symptom list, and Florida Senate Bill 1718 (S.B. 1718) items were selected and developed in consultation with FWAF community health workers, informed by psychosocial concerns raised by farmworkers during our prior fieldwork.

Farmworkers in Florida also face significant healthcare access challenges that have been compounded by recent legislative developments. Since taking effect on July 1st, 2023, S.B. 1718 has raised concerns about increasing barriers to healthcare access (32). Florida law requires hospitals that accept Medicaid to inquire about a patient’s lawful presence in the U.S. and mandates that each hospital generate a quarterly report detailing medical expenses for undocumented immigrants (33). S.B. 1718 requires private employers with over 25 employees to use E-Verify. This online verification system verifies eligibility to work lawfully in the U.S., potentially creating employment precarity that may be associated with psychological distress. According to the National Agricultural Workers Survey, 42% of farmworkers lacked work authorization (6), rendering this population vulnerable to the psychosocial stressors associated with this legislation. S.B. 1718 has been criticized for increasing systematic inequalities in healthcare, with more than 80 healthcare providers denouncing the anti-immigrant bill (34, 35).

In collaboration with the Farmworker Association of Florida (FWAF), we conducted an exploratory pilot study aimed to (1) characterize the burden of depression, anxiety, and climate change psychological distress among Florida farmworkers relative to national benchmarks, (2) explore cross-sectional associations between HRI symptoms and mental health outcomes, and (3) examine cross-sectional associations between perceived impacts of S.B. 1718 and mental health outcomes.

## Methods

### Study design and ethics approval

In collaboration with FWAF we conducted a cross-sectional, pilot survey study. The Institutional Review Board at Emory University provided approval (STUDY00007967) for the study, and all participants provided informed consent.

### Population and recruitment

Field data collection occurred at FWAF offices in the Florida towns of Apopka and Pierson in July of 2024. A convenience sample of 53 agricultural worker participants were enrolled in this study. Community health workers (CHW) recruited eligible participants during community events and farmworkers who visited FWAF offices for services provided by the association. Eligibility criteria included farmworkers aged 18 years or older employed in agricultural settings for at least 1 month in Florida. Exclusion criteria included individuals younger than 18 years of age or cognitively impaired. CHWs obtained consent from farmworkers. Consented participants completed the survey with the assistance of a FWAF CHW, who verbally administered each question in Spanish and entered participant responses directly into REDCap secure survey response platform (36). This approach was used to support participants with varying literacy levels and has been the preferred method of questionnaire administration in our prior studies with farmworker populations. All data collection was completed in a private office at FWAF at the end of the participant’s workday. At the end of the survey, study participants received a $50 gift card for participation.

### Data collection and measures

#### Demographic and work data

Bilingual study team members verbally administered a socio-demographic and general work-related survey, entering the responses in the REDCap platform (36). Participants were asked demographic questions covering age, sex, nationality, and years of education, marital status, health insurance status, primary language, and household composition. Work-related items included workplace type (nursery, fernery, other), hours worked in agriculture on the study day, years worked in U.S. agriculture, lunch break, number of additional breaks, and primary crops worked. In consultation with FWAF and in consideration of participant safety in the current Florida policy climate, direct immigration status was not asked.

#### Mental health scales

The Patient Health Questionnaire (PHQ-9) was administered to measure depression. The PHQ-9 is a nine-item screening questionnaire that asks participants how often, over the past 2 weeks, they have been bothered by each listed symptom in the PHQ-9, with responses of 0 = not at all, 1 = several days, 2 = more than half the days, and 3 = nearly every day. Total scores range from 0 to 27. A score of 10 or higher was categorized as moderate to severe depression (37). To assess anxiety, the Generalized Anxiety Disorder (GAD-7) questionnaire was administered. The GAD-7 has a total of 7 questions and uses a 0–3 response format (0 = not at all, 1 = several days, 2 = more than half the days, 3 = nearly every day) with a two-week look-back period (38). A score of 10 or higher was used to indicate moderate to severe anxiety (38). The GAD-7 and PHQ-9 have been validated for the U. S. Spanish-speaking population (39–41). The dichotomization at the ≥10 threshold for both the PHQ-9 and GAD-7 follows the established clinical screening cut-point at which further evaluation is typically recommended. This threshold also permits comparison with published studies using the same cut-point. In the present sample, the Cronbach’s alpha was 0.804 for the PHQ-9 and 0.824 for the GAD-7.

#### Florida senate bill 1718

In addition to standard psychological scales, we assessed self-reported perceptions of S. B. 1718 on this population using the two investigator-developed items administered verbally in Spanish by a bilingual CHW: 1) Have you or someone you know felt discouraged from seeking medical attention at a hospital due to being asked about immigration status? 2) Has the expansion of E-Verify made it harder to find jobs in agricultural work?

#### Climate change and mental health

The Climate Change Psychological Distress scale was used to measure climate change-related anxiety and depression (42). CCPD questions included: “Over the last 2 weeks, how often have you been bothered by the following problems?” in which there are four statements: 1) feeling nervous, anxious, or on edge because of global warming; 2) not being able to stop or control worrying about global warming; 3) little interest or pleasure in doing things because of global warming; and 4) feeling down, depressed, or hopeless because of global warming. Participants selected 0 = not at all, 1 = several days, 2 = more than half the days, and 3 = nearly every day. According to the CCPD scale, total scores across the four items are summed, and a score of 6 or higher indicates moderate to severe climate change-related anxiety and depression (42). Participants who did not understand the term “global warming” were provided an explanation using the terms “intensifying heat,” “rising temperature,” or “climate change.” In the present sample, the Cronbach’s alpha for the CCPD was 0.801.

#### Environmental temperatures

We assessed relative humidity and temperature for each participant’s workday through the Florida Automated Weather Network (FAWN) (43). The FAWN network is a 42-station network throughout mainland Florida that gathers temperature readings every 15 min. A daily average heat index was calculated using the Lu and Romps extended heat index algorithm based on the hours worked by each participant (44).

#### Heat-related symptoms

Farmworkers completed the questionnaire following their work shift and were asked to report common HRI symptoms experienced during that workday. A list of eight symptoms was presented: heavy sweating, headache, nausea, vomiting, dizziness, confusion, muscle cramps, and fainting. Participants were also asked to report any additional symptoms not specified. The total number of HRI symptoms reported was summed to generate a composite symptom count. This self-reported questionnaire of HRI symptoms has been utilized in several studies with farmworkers in Florida (3, 8, 30).

#### Statistical analysis

Means, medians, quartiles, standard deviations, and percentages were calculated to describe demographic and clinical characteristics and occupational background variables. PHQ-9, GAD-7, and CCPD scores were analyzed both as continuous summed scores and as binary indicators using the published thresholds of ≥10 for PHQ-9 and GAD-7 and ≥6 for CCPD (37, 38, 42). The number of HRI symptoms was also categorized as: none (reference), one symptom, or two or more symptoms. This categorization was chosen to balance biological plausibility (any symptom is clinically relevant; multiple concurrent symptoms indicate more pronounced heat strain). Years worked in agriculture were categorized as: 5 years or fewer, 6 to 10 years, or 11 years or more.

Missingness in the study was limited to two variables: 7 participants (13%) did not report years of education, and 2 participants (4%) did not report type of agricultural work. We used complete-case analysis for all variables, consistent with current best-practice guidance (45).

The two-sample *t*-test and one-way ANOVA were used to analyze statistically significant differences in questionnaire scores across demographic, clinical, and occupational categories. Fisher’s exact test assessed the association between severity categories of questionnaire scores and demographic, clinical, and occupational categories. Multivariable linear and logistic regression models were used to examine associations between mental health outcomes and the number of HRI symptoms, adjusting for age, sex, and years in agriculture. A *p*-value below 0.05 was considered a statistically significant result in all group comparisons. All data analyses were performed using R and R studio (46, 47).

## Results

### Demographics of study sample

We collected the data for this study during July 2024. Fifty-three participants (58% female and 42% male) enrolled in the study (Table 1) with 55% having married/coupled status. The median age of participants was 40, with a median of 8 years of education.

**Table 1:** Study sample demographic characteristics of Florida farmworkers (*N* = 53), July 2024.

Characteristic	Value
Age (years), median (Q1–Q3)	40 (33–50)
Sex, *n* (%)	
Female	31 (58%)
Male	22 (42%)
Marital status, *n* (%)	
Married/coupled	29 (55%)
Single/other	24 (45%)
Hispanic ethnicity, *n* (%)	52 (98%)
Nationality, *n* (%)	
Mexico	32 (60%)
Guatemala	6 (11%)
United States	5 (10%)
Other	10 (19%)
Identifies as Indigenous, *n* (%)	27 (51%)
Spanish spoken at home, *n* (%)	52 (98%)
Education (years), median (Q1–Q3)^a^	8 (5–10)

Values are *n* (%) for categorical variables and median (Q1–Q3) for continuous variables, where Q1 and Q3 are the 25th and 75th percentiles, respectively. Percentages are rounded to the nearest whole number and may not sum to 100%.

^a^Seven participants did not report years of education.

### Work characteristics

Workers labored a mean of 9 h per day with a mean workday heat index of 101.5 °F, 56% working in nurseries and 37% in ferneries (Table 2). The median years working in U.S. agriculture was 12 and 91% of workers reported taking a lunch break and 25% took no additional breaks.

**Table 2:** Study sample work characteristics of Florida farmworkers (*N* = 53), July 2024.

Characteristic	Value
Age started agricultural work (years), median (Q1–Q3)	17 (13–24)
Years in U. S. agriculture, median (Q1–Q3)	12 (3–24)
Hours worked on study day, median (Q1–Q3)	9 (6–10)
Workday heat index (°F), mean (SD)	101.5 (12.0)
Lunch break taken, *n* (%)	48 (91%)
Additional breaks during workday, *n* (%)	
None	13 (25%)
1	13 (25%)
2 or more	26 (49%)
Reason for no additional breaks (*n* = 13), *n* (%)	
Paid by the piece	4 (31%)
Employer does not provide breaks	7 (54%)
Want to leave early	2 (15%)
Type of agricultural work (*n* = 51), *n* (%)	
Nursery	29 (57%)
Fernery	19 (37%)
Other	3 (6%)

Values are median (Q1–Q3) for continuous variables except workday heat index, which is reported as mean (SD); values for categorical variables are n (%). Where denominators differ from *N* = 53 due to skip patterns or missing data, the subgroup denominator is shown in the row label. Percentages are rounded to the nearest whole number and may not sum to 100%. Workday heat index was calculated using the extended heat-index algorithm of (44).

### Mental health status

Overall, 11 participants (21%) scored within the moderate to severe anxiety range. Sixteen percent of female participants scored in the moderate to severe anxiety range, compared to 27% of male participants; however, these differences were not statistically significant. Moderate to severe anxiety (GAD-7 score ≥ 10) was observed at similar rates among married and single participants, with 21% in each group. Participants with less than 5 years of experience in U.S. agriculture scored an average of 5.84 (mild anxiety) on the GAD-7, while participants with 11 years or more experience scored 3.46 (minimal anxiety) but these differences were not found to be statistically significant.

Overall, 13 participants (25%) scored within the moderate to severe depression range. Twenty-nine percent of female participants scored in the moderate to severe depression range, compared to 18% of male participants; however, these differences were not statistically significant (Table 3). No difference was found in the likelihood that uninsured individuals would have higher depressive symptoms than insured individuals.

**Table 3:** Depression (PHQ-9) scores by demographic and work characteristics among Florida farmworker, July 2024 (*N* = 53).

Characteristic	Continuous PHQ-9 score			Moderate-to-severe depression (PHQ-9 ≥ 10)		
	Mean	SD	*p*	*n*	%	*p*
Sex						
Female	6.41	5.55	0.184	9	29	0.348
Male	4.45	4.82		4	18	
Marital status						
Married/coupled	5.21	5.36	0.602	6	21	0.529
Single	6.00	5.28		7	29	
Health insurance						
Yes	4.86	4.23	0.390	5	23	0.755
No	6.10	5.98		8	26	
Typically takes additional breaks at work						
Yes	4.66	4.89	0.075	7	18	0.055
No	8.25	5.93		6	46	
Years in U. S. agriculture						
≤5 years	8.16	5.65	**0.011**	9	47	**0.021**
6–10 years	1.83	3.13		0	0	
≥11 years	4.54	4.62		4	14	

PHQ-9, Patient health questionnaire-9; SD, standard deviation. Continuous PHQ-9 scores are reported as mean (SD); moderate-to-severe depression is defined as PHQ-9 ≥ 10. *p*-values are from two-sample t-tests and one-way ANOVA for continuous outcomes and from Fisher’s exact tests for categorical outcomes; significant *p*-values are shown in bold. Percentages are rounded to the nearest whole number and may not sum to 100%.

Participants with ≤ 5 years working in U.S. agriculture reported moderate to severe depression (47%), compared to those with 11 or more years (14%) (Fisher’s exact test *p* = 0.021). A statistically significant difference in mean PHQ-9 scores was observed across workers with ≤5 years (8.16), 6–10 years (1.83), and ≥11 years of experience in U.S. agriculture (4.54) (ANOVA = 4.95; *p* = 0.011).

### Heat-related illness symptoms and mental health

Of the 53 participants, 25 (47%) reported no HRI symptoms during the workday, 17 (32%) reported one HRI symptom, and 11 (21%) reported two or more HRI symptoms. Table 4 shows associations between the number of HRI symptoms reported and anxiety (GAD-7), depression (PHQ-9), and climate change psychological distress (CCPD). There was a statistically significant difference (ANOVA = 3.78; *p* = 0.030) in GAD-7 mean scores between participants with two or more HRI symptoms (7.45), participants with one HRI symptom (3.47), and those with no symptoms (3.24). Participants with two or more HRI symptoms had a depression score of 10.10, while those with one HRI symptom a score of 4.88, and participants with no HRI symptoms a score of 4.17; a statistically significant difference (ANOVA = 5.50; *p* = 0.007) was observed between groups. Additionally, those who reported two or more HRI symptoms had the highest moderate to severe levels of depression (55%), compared to participants with one (18%) and no HRI symptoms (16%); a significant association between the number of HRI symptoms and levels of depression (Fisher’s exact test *p* = 0.035). The mean CCPD scores were 3.94 among females and 3.32 among males, reflecting mild levels of climate change psychological distress; however, this difference was not statistically significant. Participants reporting two or more HRI symptoms exhibited a higher prevalence of moderate to severe climate change psychological distress (55%) compared to those reporting one (35%) or no symptoms (12%), indicating a significant association between the number of HRI symptoms and the severity of climate change psychological distress (Fisher’s exact test *p* = 0.025).

**Table 4:** Mental health outcomes by number of heat-related illness (HRI) symptoms among Florida farmworkers (*N* = 53), July 2024.

Outcome and HRI symptoms	Continuous score			Moderate-to-severe		
	Mean	SD	*p*	*n*	%	*p*
Anxiety (GAD-7)						
0 symptoms	3.24	3.82	**0.030**	4	16	0.367
1 symptom	3.47	4.27		3	18	
≥2 symptoms	7.45	5.84		4	36	
Depression (PHQ-9)						
0 symptoms	4.17	5.02	**0.007**	4	16	**0.035**
1 symptom	4.88	3.89		3	18	
≥2 symptoms	10.10	5.92		6	55	
Climate change psychological distress (CCPD)						
0 symptoms	2.72	2.70	0.068	3	12	**0.025**
1 symptom	4.00	3.69		6	35	
≥2 symptoms	5.36	3.14		6	55	

GAD-7, Generalized anxiety disorder-7; PHQ-9, Patient health questionnaire-9; CCPD, Climate change psychological distress; SD, standard deviation. Continuous scores are reported as mean (SD); moderate-to-severe categories are defined as GAD-7 ≥ 10, PHQ-9 ≥ 10, and CCPD ≥ 6. *p*-values are from one-way ANOVA for continuous outcomes and from Fisher’s exact tests for categorical outcomes; significant *p*-values are shown in bold.Percentages are rounded to the nearest whole number and may not sum to 100%.

### HRI and mental health: multivariable findings

The multivariable regression estimates below are presented as exploratory signal detection rather than as precise effect estimation. In multivariable linear regression models, participants with ≥ 2 HRI symptoms had a 4-point higher mean GAD-7 score (95% CI: 0.70–7.29; *p* = 0.019) and a 5-point increase in mean PHQ-9 score (95% CI: 1.30–8.68; *p* = 0.009) compared to those with no symptoms. They also had a 2-point higher mean CCPD score, although the confidence interval included the null (95% CI: −0.27–4.42; *p* = 0.082).

For logistic regression analyses, mental health categories (“minimal to mild” vs. “moderate to severe”) were used as binary outcomes. Participants with ≥ 2 HRI symptoms had higher odds of depression (OR: 8.48; 95% CI: 1.29–73.26; *p* = 0.034) and climate change psychological distress (OR: 10.07; 95% CI: 1.69–77.83; *p* = 0.016) compared to those with no symptoms. They also had higher odds of moderate to severe anxiety (OR: 6.23; 95% CI: 0.81–65.35; *p* = 0.091) although this estimate was not statistically significant. Substantial uncertainty across models is reflected in the wide confidence intervals. These patterns are consistent with Fisher’s exact test results (Table 4).

### Florida senate bill 1718

Participants who reported feeling discouraged from seeking hospital care due to S.B. 1718 exhibited higher mean depression scores (PHQ-9 = 6.59) compared to those who did not feel discouraged (PHQ-9 = 3.00), a difference that was statistically significant (*t* = 3.07; two-tailed *p* = 0.008). No statistically significant associations were observed between S.B. 1718 (feeling discouraged from seeking hospital care in response to immigration status inquiries or E-Verify implementation) and levels of anxiety (GAD-7) or climate change psychological distress (CCPD).

## Discussion

This exploratory pilot study aimed to characterize the burden of depression, anxiety, and climate change psychological distress among Florida farmworkers and to explore cross-sectional patterns between mental health outcomes, HRI symptoms, and perceived impacts of S.B. 1718. Within the constraints of an exploratory pilot study, we observed elevated prevalence of rates of anxiety, depression, and climate change psychological distress relative to national benchmarks, along with cross-sectional associations between HRI symptom burden and all three mental health outcomes. These findings contribute to the growing body of literature on occupational heat exposure and mental health and serve as an important foundation for future, large-scale investigations.

### Mental health status

The rates of anxiety observed in our sample were notably higher than both national benchmarks and those reported in prior farmworker studies, a pattern that likely reflects the compounding vulnerabilities unique to this population. Nationally, 6.7% of U.S. adults report moderate to severe anxiety and 6.2% of Hispanic adults (22). Among agricultural populations, 12.5% of 1,001 farmworkers in California reported anxiety symptoms (48). In North Carolina, a sample of 69 of farmworkers reported 16% to have moderate to severe anxiety (13). When examining mean GAD-7 scores, our sample scored 4.19 (95% CI: 2.90–5.47), slightly higher than the 3.54 reported among 241 Midwestern farmworkers (49), and 4.12 among 58 farmworkers in California (50).

In addition to elevated anxiety, 25% (95% CI: 14–38%) of our study participants met the criteria for moderate to severe depression, with a mean PHQ-9 score of 5.59 (95% CI: 4.08–7.06). The prevalence of moderate to severe depression and mean PHQ-9 score exceed both national statistics and those reported for farmworkers. Data from the National Health Interview Survey reported moderate to severe depression in 7.5% of adults and 7% of Hispanic adults (22). Compared to other agricultural populations, our findings reveal similarly elevated depression rates. Studies of farmworkers have documented varying but consistently high depression prevalence: 46% of 200 farmworkers in Nebraska were categorized as depressed (10), while 11% of 150 farmworkers in North Carolina met depression criteria (51). Among California farmworkers, the mean PHQ-9 score was 4.76 (52), lower than our sample’s mean of 5.59.

Direct comparisons across these studies should be interpreted cautiously. The samples differ in region, crop type, season of data collection, recruitment setting (advocacy organization, clinic, or community-based sampling), and administration mode, all of which plausibly contribute to observed divergences. For example, our July data collection captured peak heat exposure, whereas some comparison studies span a broader seasonal window.

The elevated depression burden among newer farmworkers warrants targeted attention in future research. These results are likely attributable to multiple stressors affecting newer farmworkers. One potential explanation is acculturative stress, which may arise as individuals adjust to a new cultural environment while simultaneously adapting to the demanding physical conditions of agricultural work (49). However, acculturation was not directly measured in this study, and its role should be considered a hypothesis for future investigations rather than an explanation of the present findings. The combination of potential cultural adaptation challenges and exposure to harsh working conditions, including extreme weather and high-intensity labor, may compound psychological distress among farmworkers. Conditions during our study period reflect this reality: the mean heat index during workdays was 101.5 °F, and 50% of participants reported having one or fewer breaks in addition to lunch during shifts averaging 9 h. As such, mental health in this population cannot be disentangled from the physical conditions of work.

We also observed a non-monotonic relationship between tenure and depressive symptoms, with PHQ-9 scores highest in the ≤5 year group, lowest in the 6–10 year group, and intermediate in the ≥11 year group. This pattern is broadly consistent with a “healthy worker survivor effect,” whereby more highly distressed workers are more likely to exit the workforce over time, such that longer-tenured workers may reflect a selected population with greater retention and cumulative exposure (53).

### Heat-related illness symptoms and mental health

The high prevalence of CCPD in our sample is striking and extends beyond national benchmarks, where only 2% of the U.S population and 9% of Hispanic adults report moderate to severe CCPD (42). However, the elevated CCPD scores should be interpreted cautiously. Because participants who required clarification of ‘global warming’ were provided alternative terms (e.g., “intensifying heat,” “rising temperature”), the elevated CCPD scores in our sample may partly reflect distress about proximal occupational heat exposure rather than abstract climate concern. These two interpretations cannot be separated without direct disaggregation.

As temperatures continue to rise, farmworkers face persistent exposure to extreme heat and humidity during their workday, which are conditions that are particularly characteristic of Florida’s agricultural environment. In a study in the rural U.S. Mexico border region, farmworkers reported that weather was a significant perceived stressor due to their constant exposure to extreme weather conditions while working (54). Emerging studies have reported links between occupational and environmental heat exposure and adverse mental health outcomes among workers (55). However, the mechanisms of action are not fully understood and likely involve an interplay between physiological, cultural, environment, policy and social factors that may be exacerbated by high temperatures (55, 56).

Notably, participants who reported two or more HRI symptoms during their workday had significantly higher mental health scores compared to those reporting one or no symptoms, as well as higher rates of moderate to severe depression. Just as strenuous work in high ambient temperatures can increase physiological strain and complicate chronic conditions such as diabetes and hypertension (29), it may also contribute to mental health conditions such as anxiety and depression (29, 55).

### Florida senate bill 1718

The policy environment in Florida has created additional stressors for immigrant farmworkers through increasingly restrictive legislation. The 2023 S.B. 1718 requires hospitals to inquire about patients’ immigration status and mandates E-Verify usage for employment (33). The impact of these policies on immigrant communities has been substantial. A University of South Florida study found that 79% of non-U.S citizens reported that S.B. 1718 made their lives more difficult, with 74% deterred from seeking medical services when needed (57). Mental health consequences were equally pronounced: 68% reported increased psychological stress for themselves and their families, with 64% experiencing moderate to severe distress (57). Our observations are consistent with this broader pattern: participants who reported feeling discouraged from seeking hospital care due to immigration status inquiries had significantly higher depression scores compared to those who did not.

Our findings can be considered in relation to a pre-pandemic study of Florida farmworkers that reported lower mental health burden among 80 participants in 2019–2020 (58). However, the studies are not directly comparable due to differences in recruitment, sample composition, and measurement context, and no inference about temporal change can be made. Differences in reported outcomes may reflect these methodological factors as well as broader contextual stressors, including the COVID-19 pandemic, immigration policy changes (e.g., S.B. 1718), and sociopolitical conditions. Longitudinal research is needed to evaluate trends over time. These findings are consistent with prior literature suggesting associations between restrictive immigration policies and mental health and healthcare access among immigrant populations (59, 60).

### Limitations

Limitations should be acknowledged. First, convenience sampling through FWAF offices introduces potential selection bias, as individuals engaged with service or advocacy organizations may differ from the broader farmworker population in psychosocial vulnerability, immigration-related awareness, and willingness to report distress. Second, while the sample size (*n* = 53) was appropriate for a pilot study, it limits statistical power and contributes to wide confidence intervals. Third, several important variables, including acculturation, immigration status, food insecurity, and housing insecurity, were not measured and therefore could not be included in adjusted models, raising the possibility of unmeasured confounding. Fourth, all outcomes were based on self-reported screening instruments rather than clinical diagnoses and may be affected by recall and social desirability bias. Although the PHQ-9 and GAD-7 are validated screening tools, they do not by themselves establish a clinical diagnosis. The CCPD scale has not been validated in farmworker populations and the alternative terminology provided to participants who did not understand ‘global warming’ may have shifted the construct being measured; CCPD findings should therefore be interpreted as exploratory. Finally, the cross-sectional design precludes causal inference or assessment of temporal ordering. Together, these limitations underscore that findings are hypothesis-generating rather than confirmatory.

Future research should employ longitudinal designs to examine farmworkers and other outdoor workers in climate-sensitive industries who face chronic exposure to high ambient temperatures. Such studies are essential for elucidating the mechanisms by which extreme heat affects mental health in the context of high global temperatures. Research must assess the complex interplay between physiological, cultural, environmental, policy, and social factors to inform the development of tailored interventions to protect the mental health of workers in climate-sensitive sectors.

### Conclusion

This pilot study provides preliminary evidence of an elevated burden of anxiety, depression, and climate change psychological distress among farmworkers in Florida, particularly among those reporting two or more HRI symptoms. The findings suggest an association between occupational and environmental heat exposure indicators and adverse mental health outcomes in a climate-sensitive industry already burdened by significant heat strain. These findings should also be understood within a broader structural context, including ongoing immigration policy changes such as S.B. 1718, which may shape lived experiences of stress and access to care among farmworker populations. Longitudinal research is needed to clarify the temporal and causal relationships among heat exposure, policy environment, and mental health outcomes, and intervention research is needed to evaluate strategies for protecting mental health in this climate-sensitive occupational population.

## Data Availability

The raw data supporting the conclusions of this article will be made available by the authors, without undue reservation.
